# Vitamin A deficiency and vitamin A supplementation affect innate and T cell immune responses to rotavirus A infection in a conventional sow model

**DOI:** 10.3389/fimmu.2023.1188757

**Published:** 2023-04-25

**Authors:** Juliet Chepngeno, Joshua O. Amimo, Husheem Michael, Sergei A. Raev, Kwonil Jung, Marcia V. Lee, Debasu Damtie, Alfred Omwando, Anastasia N. Vlasova, Linda J. Saif

**Affiliations:** ^1^ Center for Food Animal Health, Department of Animal Sciences, College of Food Agricultural and Environmental Sciences, The Ohio State University, Wooster, OH, United States; ^2^ Department of Veterinary Preventive Medicine, The College of Veterinary Medicine, The Ohio State University, Columbus, OH, United States; ^3^ Department of Animal Production, Faculty of Veterinary Medicine, University of Nairobi, Nairobi, Kenya; ^4^ Department of Immunology and Molecular Biology, School of Biomedical and Laboratory Sciences, College of Medicine and Health Sciences, University of Gondar, Gondar, Ethiopia; ^5^ The Ohio State University Global One Health LLC, Eastern Africa Regional Office, Addis Ababa, Ethiopia; ^6^ Department of Public Health, Pharmacology and Toxicology, Faculty of Veterinary Medicine, University of Nairobi, Nairobi, Kenya

**Keywords:** Rotavirus A, innate immune responses, dendritic cells, natural killer cells, T cells, sow model, maternal passive immunity

## Abstract

Rotavirus A (RVA) causes ~200,000 diarrheal deaths annually in children <5yrs, mostly in low- and middle-income countries. Risk factors include nutritional status, social factors, breastfeeding status, and immunodeficiency. We evaluated the effects of vitamin A (VA) deficiency/VA supplementation and RVA exposure (anamnestic) on innate and T cell immune responses in RVA seropositive pregnant and lactating sows and passive protection of their piglets post-RVA challenge. Sows were fed VA deficient (VAD) or sufficient (VAS) diets starting at gestation day (GD)30. A subset of VAD sows received VA supplementation from GD|76 (30,000IU/day, VAD+VA). Sows (6 groups) were inoculated with porcine RVA G5P[7] (OSU strain) or Minimal Essential Medium (mock) at GD~90: VAD+RVA; VAS+RVA; VAD+VA+RVA; VAD-mock; VAS-mock; and VAD+VA-mock. Blood, milk, and gut-associated tissues were collected from sows at several time points to examine innate [natural killer (NK), dendritic (DC) cells], T cell responses and changes in genes involved in the gut-mammary gland (MG)-immunological axis trafficking. Clinical signs of RVA were evaluated post inoculation of sows and post-challenge of piglets. We observed decreased frequencies of NK cells, total and MHCII^+^ plasmacytoid DCs, conventional DCs, CD103^+^ DCs and CD4^+^/CD8^+^ and T regulatory cells (Tregs) and NK cell activity in VAD+RVA sows. Polymeric Ig receptor and retinoic acid receptor alpha (RARα) genes were downregulated in mesenteric lymph nodes and ileum of VAD+RVA sows. Interestingly, RVA-specific IFN-γ producing CD4^+^/CD8^+^ T cells were increased in VAD-Mock sows, coinciding with increased IL-22 suggesting inflammation in these sows. VA supplementation to VAD+RVA sows restored frequencies of NK cells and pDCs, and NK activity, but not tissue cDCs and blood Tregs. In conclusion, similar to our recent observations of decreased B cell responses in VAD sows that led to decreased passive immune protection of their piglets, VAD impaired innate and T cell responses in sows, while VA supplementation to VAD sows restored some, but not all responses. Our data reiterate the importance of maintaining adequate VA levels and RVA immunization in pregnant and lactating mothers to achieve optimal immune responses, efficient function of the gut-MG-immune cell-axis and to improve passive protection of their piglets.

## 1 Introduction

Rotavirus (RV) is the major cause of severe, acute gastroenteritis in young animals and children worldwide (1). RV diarrheal disease in swine is associated with weight loss, morbidity and mortality causing major losses to the pork industry. Specifically, RV species A (RVA) causes severe diarrhea in weaned piglets (2–5). RVA infects children in both developed and developing countries alike; however, children <5years of age in developing countries experience severe clinical symptoms (6). Development of human RVA vaccines and their introduction into national vaccination programs more than two decades ago led to a reduction in RVA burden in developed countries (7). However, the World Health Organization (WHO) estimates that ~200,000 deaths annually in children <5yrs of age are related to RVA, mainly in the developing countries (8). Coincidentally, vitamin A deficiency (VAD) rates in children and pregnant women are high in these countries (9, 10). WHO estimated that ~250 million school-aged children are VAD, with ~5 million of these children showing clinical signs such as night blindness (11). VAD is associated with increased susceptibility to diseases such as measles, diarrheal diseases and higher mortality rates in the affected populations (12, 13).

Vitamin A (VA) is not synthesized in the body *de novo*; therefore, it must be acquired through diet as preformed VA (retinol and retinyl ester from animal sources) or pro-VA (beta-carotenoids from colorful fruits and vegetables). VA encompasses a family of retinoids; retinal (retinaldehyde), retinol, and retinoic acid (RA). These retinoids are important for various functions in the body including: cell differentiation, erythrocyte production, reproduction, epithelial surface integrity and normal immune responses (14, 15). RA is the most important biologically active VA metabolite that has been shown to directly modulate both innate and adaptive immunity in the mucosal tissues of the body leading to immune responses in presence of pathogens or maintaining homeostasis in absence of pathogens (16–18).

Important cellular components of the innate immunity system comprise natural killer (NK) cells, dendritic cells (DCs) and other cells that aid in pathogen clearance and play a role in initiating adaptive immune responses. NK cells are cytotoxic cells that eliminate virus infected and tumor cells, limiting spread of infections or malignancies (19). Radaeva and colleagues evaluated the effects of RA on NK cells in murine stellate cells and discovered that NK cell numbers and activity were upregulated by RA through the RA early inducible (RAE) ligand upregulation (20), while Dawson et al., found that in peripheral blood mononuclear cells (PBMC) of rats, marginal VAD decreased NK frequency and activity of these cells (21). Furthermore, Zhao and colleagues showed that VAD decreased NK cell function in spleens of rats (22). Previous studies of mice have demonstrated that VA supplementation increased NK cell activity in different *in vitro* and *in vivo* experiments (23, 24). Taken together, these studies suggest that VA plays a key role in regulating NK cell numbers and activity.

Intestinal DCs sample gut luminal contents, surveying for invading pathogens and play a key role in bridging the innate and the adaptive immune systems by processing and presenting antigens to naïve T cells *via* major histocompatibility complex (MHC) (25). This leads to the activation of B cell and cell-mediated immune responses, eliciting specific pathogen elimination and memory responses (26). Using a mouse model, studies have shown that RA is necessary for differentiation of DCs in the mucosa which could contribute to clinical abnormalities observed in VAD mice (16). Apart from RA regulating DC differentiation, intestinal CD103^+^ DCs express enzymes which metabolize VA into RA, making it available in the gut mucosa for imprinting gut-homing characteristics to effector T and B cells (27–29). In addition, CD103^+^ DCs are important in induction of oral tolerance and maintenance of homeostasis in the gut. For instance, in the presence of RA and transforming growth factor β (TGF-β), CD103^+^ DCs induce T regulatory cells (Tregs) differentiation in the gut leading to oral tolerance. In addition, a study using a human enteroid system showed that RA induced differentiation of human intestinal microfold cells through lymphotoxin signaling pathway (30).

Lopez-Guerrero and colleagues studied the role of DCs in mice infected with wild-type murine RVA. They observed that 48h after RVA infection, DCs from the Peyer’s patches migrated to the dome area with increased surface activation markers on these DCs (31). In another study, Rosales-Martinez and colleagues generated DCs from human umbilical cord monocytes and adult peripheral blood monocytes and infected these cells with RVA. They found that DCs derived from neonates induced activation and strong CD4^+^ T cell responses when treated with RVA just like adult DCs; however, adult DCs were capable of inducing anti-inflammatory cytokines (Interleukin [IL]-10 and TGF-β) in contrast to the neonatal DCs (32). Furthermore, Deal and colleagues determined the effect of RA on plasmacytoid DCs (pDCs) isolated from human blood and observed that RA treatment led to pDCs maturation and activation resulting in production of type 1 IFN and other pro-inflammatory cytokines (33). We have previously shown that DCs play an important role during RVA infection in a gnotobiotic (Gn) pig model and that pDCs are the main source of type 1 IFN (34). Moreover, we have previously shown that prenatally-acquired VAD in swine skewed innate immune responses indicated by increasing numbers of total DCs and decreasing frequencies of CD103^+^ DCs in both RVA- and mock-challenged Gn piglets (35). However, Gn pigs lack gut microbiome which has been shown to interact with RA to induce homeostasis in the gut (36). In addition, recent studies suggest that microbiota produce retinaldehyde dehydrogenase that converts retinal to RA and produces high concentration of the active retinoids including RA, which primes host immune responses (37–40). Recently, we have shown that VAD impairs B cell responses in pregnant and lactating conventional sows and passive protection of their piglets against RVA while oral VA supplementation and RVA immunization of these sows improved B cell responses and protection of their piglets against virulent RVA (41). The role of VAD and VA supplementation on innate and T cell immune responses necessary to initiate adequate B cell and passive responses to infections is not well understood. Therefore, the aim of our study was to determine the effects of VAD and VA supplementation on innate and T cell immune responses in conventional pregnant and lactating sow model of RVA infection.

## 2 Materials and methods

### 2.1 Virus

RVA OSU (G5P[7]) virus pool was obtained by inoculating 3-day old Gn piglets with virulent RVA OSU. The small and large intestinal contents obtained 3 days post inoculation were tested for sterility and stored at -80°C until use. Cell culture immunofluorescence (CCIF) assay was used to determine the virus titer. The virus was used to inoculate RVA seropositive sows (Mimic maternal immunization) at a dose of 1 × 10^9^ fluorescent focus units (FFU) per sow diluted in Minimum Essential Media [MEM (Life Technologies, Carlsbad, CA, USA)]. Piglets were inoculated with the same virus at a dose of 1 × 10^8^ FFU per piglet.

### 2.2 Animals and experimental design

**Figure 1:**
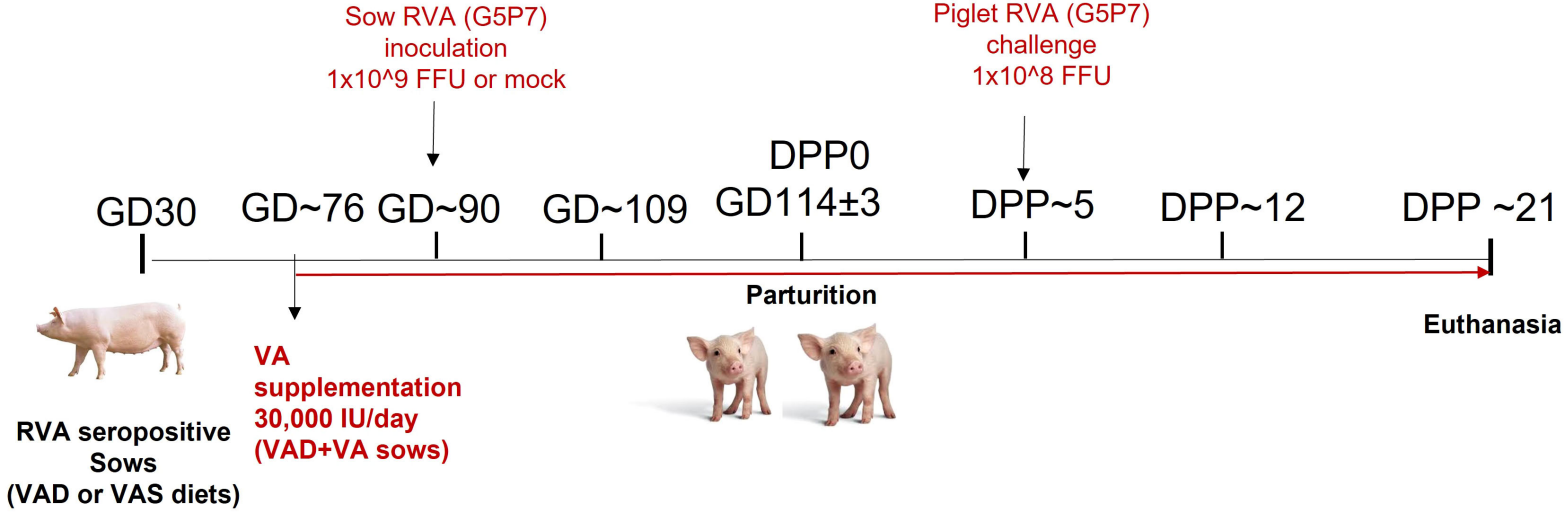
Experimental design to evaluate the effects of vitamin A deficiency/vitamin A supplementation and RVA inoculation (anamnestic response) on innate and T cell responses of RVA seropositive pregnant sows and passive protection of their piglets. (GD, Gestation Day; DPP, Day Post Partum; VAS, Vitamin A sufficient diet; VAD, Vitamin A deficient diet; VA, Vitamin A; RVA, Rotavirus A).

All animal experiments were approved by the Institutional Animal Care and Use Committee at The Ohio State University. All methods were carried out in accordance with our approved protocol and optimized regulations were followed. Sample collection and euthanasia were carried out humanely. Pregnant RVA seropositive sows (Landrace × Yorkshire × Duroc cross-bred) of parity 3-5 were obtained from The Ohio State University swine center facility at gestation day (GD) 30 and housed individually in pens. Sows were randomly assigned to one of the six treatment groups (1): VAD+RVA (*n* = 4) (2); VAS+RVA (*n* = 3); (3) VAD+VA+RVA (*n* = 4); (4) VAD-mock (*n* = 3) (5) VAS-mock (*n* = 4); and (6) VAD+VA-mock (*n* = 3). Sows were fed either VAD or VAS diet and a subset of VAD fed sows were supplemented with VA (30,000 IU per day) orally starting at GD~76 to the end of experiment (**Figure 1**). Sows were orally inoculated with RVA OSU strain or MEM (mock) at GD~90 and allowed to farrow naturally at GD114 (± 3). All piglets were orally challenged with RVA OSU strain at day postpartum (DPP)~5 and sampled as describe below (41).

Blood samples were collected from sows at GD~90, GD~109, days postpartum (DPP)~5, DPP~12 and DPP~21 for peripheral mononuclear cell (PMNC) isolation (41). Rectal swab samples were collected every other day following RVA inoculation to examine RVA RNA shedding and to record fecal consistency for diarrhea scores (41). After farrowing, colostrum was collected within 24h of parturition. Colostrum (at DPP0) and milk (at DPP ~5, ~12 and ~21) were collected after administration of 3 ml oxytocin intramuscularly for milk letdown (41). Sow spleens, mesenteric lymph nodes (MLNs), mammary glands (MG) and ileum were collected at euthanasia (DPP~21) for MNCs isolation (41). Piglets were challenged at DPP~5 and RVA RNA shedding, and fecal consistency were determined from rectal swabs collected after challenge.

### 2.3 Fecal consistency scores and RVA RNA quantification using RT-qPCR

Rectal swab samples were collected from sows from post inoculation day (PID)0 to PID12 and from both sows and piglets post-piglet challenge day (PCD0-PCD12) for examining RVA RNA shedding titers and diarrhea (41). Fecal consistency was scored as follows: 0, normal; 1, pasty/semi-liquid; 2, liquid; pigs with fecal scores more than 2 were considered diarrheic. Rectal swabs were suspended in 2 mL of MEM (Life Technologies, Waltham, MA, USA) supplemented with antibiotics/antimycotic, and clarified by centrifugation at 800× *g* for 10 min at 4°C. RNA was extracted using 50µL of the sample and MagMAX kit following the manufacturer’s instructions (Applied Biosystems, Foster City, CA USA). RT-qPCR was used to determine RVA RNA titers using primers; forward 5′-GCT AGG GAY AAA ATT GTT GAA GGT A-3′, reverse 5′-ATT GGC AAA TTT CCT ATT CCT CC-3′ and hydrolysis probe 5′-FAM-ATG AAT GGA AAT GAY TTT CAA AC-MGB-3′ (42) and Qiagen one step RT-PCR kit following manufacturers protocol (Qiagen, Germantown, MD USA).

### 2.4 Isolation of mononuclear cells

Peripheral blood MNCs (PBMC) were isolated as described previously (43). Briefly, sow peripheral blood was collected and mixed with10% citrate dextrose solution (ACD). Ficoll-Paque method was used to isolate PBMC that were then resuspended in 5ml modified Gibco Roswell Park Memorial institute 1640 medium (E-RPMI) (41). Tryptophan blue (0.002%) and a cell counter was used to determine the quantity and viability of the PBMCs (44). Isolated MNCs were resuspended in freezing medium (10% FBS and 10% DMSO) and placed in vials at 1x10^7^ cells/ml and stored in liquid nitrogen until use. Milk MNCs were processed following procedures outlined in Chepngeno et al. (41). Selected tissues [Spleen (200g), MLN (100g), MG (200g) and ileum (150g)] were obtained from all the sows at euthanasia (DPP ~21) and washed 2x in wash media [RPMI 1640 + 0.1% ampicillin +0.1%gentemicin+1%N-2-hydroxyethylpiperazine-N-2-ethane sulfonic acid (HEPES)]. Tissues were cut into 1cm x 1cm pieces and pressed through tissue collectors and the supernatant collected. MNC isolation was performed following procedures detailed in Ward et al. (41, 45). All MNCs were resuspended in freezing medium (10% FBS and 10% DMSO) and placed in vials at 1x10^7^ cells/ml and stored in liquid nitrogen until use.

### 2.5 Flow cytometry

#### 2.5.1 Staining and flow cytometry analysis to measure frequencies of NK cells, DCs, and T cells

MNCs were retrieved from liquid nitrogen, thawed, washed in 20ml E-RPMI and centrifuged at 450 x g for 10 minutes at 4°C. The supernatant was discarded and E-RPMI (2ml) was added to the cell pellet and the cell suspension was transferred to a 24 well plate (Thermofisher, Waltham, MA USA). The cells were incubated at 37°C, 5% CO_2_ overnight (41). Antibody staining for NK cells markers (SWC3a^-^ CD16^+^), markers for different subsets of DCs (pDCs- SWC3a^+^CD4^+^CD11b^-^, conventional DCs (cDCs) - SWC3a^+^CD4^-^CD11b^+,^ CD103^+^ DCs - SWC3a^+^CD4^-^CD103^+^) was performed as previously described (35, 43, 46, 47) (Antibodies and isotype controls are in **Table S1**). Antibody staining for Treg cells (natural Tregs-CD4^+^/CD8^+^CD25^+^FOXP3^+^, inducible Tregs - CD4^+^/CD8^+^CD25^−^FOXP3^+^ and activated Tregs- CD4^+^/CD8^+^CD25^+^FOXP3^−^) were performed as described previously by Chattha et al. (47, 48). To determine the frequencies of RVA-specific IFN-γ-producing CD4^+^ and CD8^+^ cells (CD3^+^, CD4^+^/CD8^+^ IFN-γ), MNCs were restimulated *in vitro* with the semi purified RVA virus (12 μg/ml) and porcine cross-reactive human CD49d monoclonal antibody (0.5 μg/ml) (clone 9F10; BD Pharmingen) for 18 h and stained as previously described (48, 49). Analysis of different cell populations was performed by acquiring 50,000 events using CFlow software on Accuri C6 plus cytometer (Accuri cytometers; BD Biosciences). Sequential gating strategies applied to determine frequencies of NK cells, pDCs, cDCs, CD103^+^ DCs, Tregs and RVA-specific IFN-γ-producing CD4^+^/CD8^+^ cells have been outlined in **Figure S1**.

#### 2.5.2 Natural killer cell cytotoxicity assay

Briefly, K562 cells [50,000 cells/ml per well] were stained with carboxyfluorescein succinimidyl ester (CFSE) using Abcam CFSE kit (Waltham, MA). CFSE labeled K562 cells were washed and incubated with isolated MNCs at the ration 1:10 (K562: MNCs) overnight at 37°C, 5% CO_2_. 7-Amino-Actinomycin D (7-AAD) (Thermofisher, Waltham, MA USA) was added to determine viable cells. Acquisition and analysis of K562 dead cells (CFSE^+^7AAD^+^) was performed by acquiring 50,000 events using CFlow software on Accuri C6 plus cytometer and analyzing using the same software. Gating strategy for NK cell cytotoxicity assay has been outlined in **Figure S1**.

### 2.6 Total RNA isolation and RT-PCR for gene expression

RNA isolation was conducted using Directzol total RNA kit (Zymogen, Irvine, CA, Cat # R2052) following the manufacturers protocol. Briefly thawed MNCs were washed twice in 1xPBS, resuspended in 500 µl of Trizol reagent and thoroughly mixed. An equal volume of ethanol (95-100%) was added, transferred into a column, and centrifuged at 12,000 x g for 30 seconds. The supernatant was discarded and 400 µl of RNA wash buffer was added and centrifuged at 12,000 x g for 30 seconds. DNase 1 mix was added into the column and incubated at RT for 15 minutes. Pre-RNA wash buffer (400µl) was added into the column and centrifuged at 12,000 x g for 30 seconds. RNA wash buffer (700µl) was added into the column, centrifuged 12,000 x g for 30 seconds before total RNA was eluted using 50µl sterile H_2_O. Quantitative RT-PCR was used to determine the expression of selected genes using an equal amount of total RNA (50 ng) with SYBR Green RT-PCR Kit (Applied Biosystems, CA USA) using gene specific primers (**Table S2**). GAPDH was used as a house keeping gene. The Delta-delta method was used to analyze the differences in gene expression among the different groups relative to the VAS+RVA group.

### 2.7 Cytokine ELISA

Interleukin -10, IL-6, IFN-α, IL-22 cytokine ELISA kits were obtained from R&D systems (Minneapolis, MN, USA -IL-10 and IL-6) and Invitrogen (Waltham, MA, USA- IFN-α and IL-22). Briefly, 96-well pre-coated plates were obtained and 100µL of assay diluent was added to all wells. Standards, controls, and serum (100µL) from sows were added to the wells and incubated in for 2.5h at room temperature in an orbital shaker. The plates were washed, and the conjugate added and incubated for 2h at room temperature on the shaker and then plates were washed. Substrate solution was added and incubated at room temperature for 30 min in the dark. Stop solution was added and the plates were analyzed by measuring absorbance at 450nm on the spectra Max 340 PC (Molecular Devices, Sunnyvale, CA).

### 2.8 Statistical analyses

The mean frequencies of NK cells, K562 dead cells %, DCs, Treg cells, IFN-γ secreting T cells, mRNA expression and cytokine levels were calculated for each treatment group using GraphPad prism 9.0 version (GraphPad Software, Inc., San Diego, CA, USA) and one-way or two-way analysis of variance (ANOVA) analysis followed by Tukey-Kramer test to determine significant differences among the groups. Statistical significance was assessed at P<0.05.

## 3 Results

### 3.1 Hepatic vitamin A levels were decreased in VAD sows and significantly decreased in their piglets

Overall serum VA levels were comparable in all treatment groups indicative of VAD sows mobilizing hepatic VA storage (**Table 1**). However, VAD sows had decreased hepatic levels compared to VAS and VA supplemented VAD sows. Piglets of VAD sows had significantly decreased hepatic VA levels at DPP~21 compared with piglets of VAS sows. VA supplementation to VAD sows significantly increased hepatic levels in their piglets (**Table 1**).

**Table 1:** Mean serum and hepatic vitamin A levels in RVA-inoculated sows at different gestation and lactation time points and in piglets (hepatic) at DPP 21.

*Treatment Group*	*GD~30*	*GD~76*	*GD~109*	*DPP~21*		*Piglets* *DPP~21*
	Serum (PPM)	Serum (PPM)	Serum (PPM)	Serum (PPM)	Hepatic (PPM)	Hepatic (PPM)
*VAD*	0.185	0.224	0.225	0.263	64	6.92^a^
*VAS*	0.185	0.242	0.228	0.260	93.75	16.33^b^
*VAD+VA*	0.193	0.228	0.210	0.252	103.33	10.75^c^

(GD, gestation day; DPP, day post-partum). Superscript letters (a, b, and c) indicate significant differences among the groups.

### 3.2 Piglets of VAD-Mock sows had the highest RVA RNA shedding levels and cumulative fecal scores

**Figure 2:**
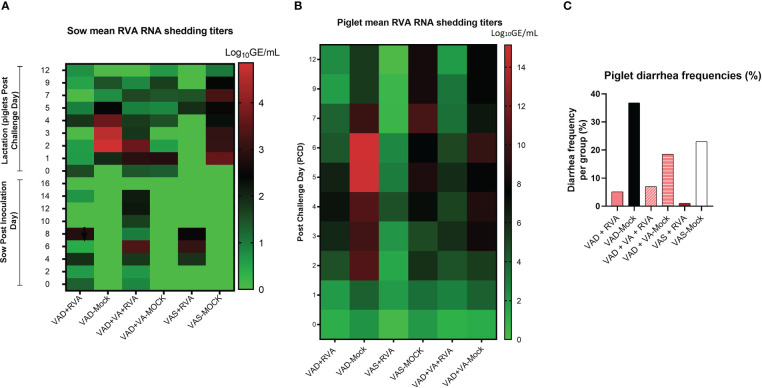
Analysis of RVA RNA shedding titers and diarrhea in sows and piglets. RVA OSU strain was used to inoculate sows at gestation day (GD)~90 while piglets were challenged at day post-partum (DDP)~5. RVA RNA titer was determined by RT-qPCR. **(A)** Heat map showing RVA RNA shedding in sows post sow inoculation (PID0-16) and post-piglet challenge (PCD0-12) expressed as log_10_ GE/mL. **(B)** Heat map depicting RNA shedding in piglets post-piglet challenge (PCD0-12) expressed as log_10_ GE/mL. **(C)** The number of piglets that developed diarrhea per group expressed as a percentage, where fecal scores were scored as follows: 0, normal; 1, pasty; 2, semiliquid; 3, liquid, and diarrhea was considered as score of ≥2. Piglets were considered diarrheic if they had a score of ≥2 on any day between PCD0 and 12.

While all RVA (but not mock) inoculated sows shed the virus post inoculation (**Figure 2A**), none of them developed diarrhea following RVA inoculation. During lactation, all mock sows had increased RVA RNA shedding after RVA challenge of their piglets (DPP~5/PCD0) compared with the sows inoculated with RVA during gestation (**Figure 2A**) (41). Overall, VAD-mock litters had the highest RVA RNA shedding throughout the experiment with highest titers at PCD6-7 (**Figure 2B**), coinciding with the highest percentage of piglets that developed diarrhea (**Figure 2C**) (41). Overall, all litters from RVA inoculated sows had decreased RVA RNA shedding regardless of the VA diet.

### 3.3 Evaluation of NK cell frequencies and function

#### 3.3.1 Vitamin A deficiency decreased the frequencies of NK cells in VAD+RVA sows

**Figure 3:**
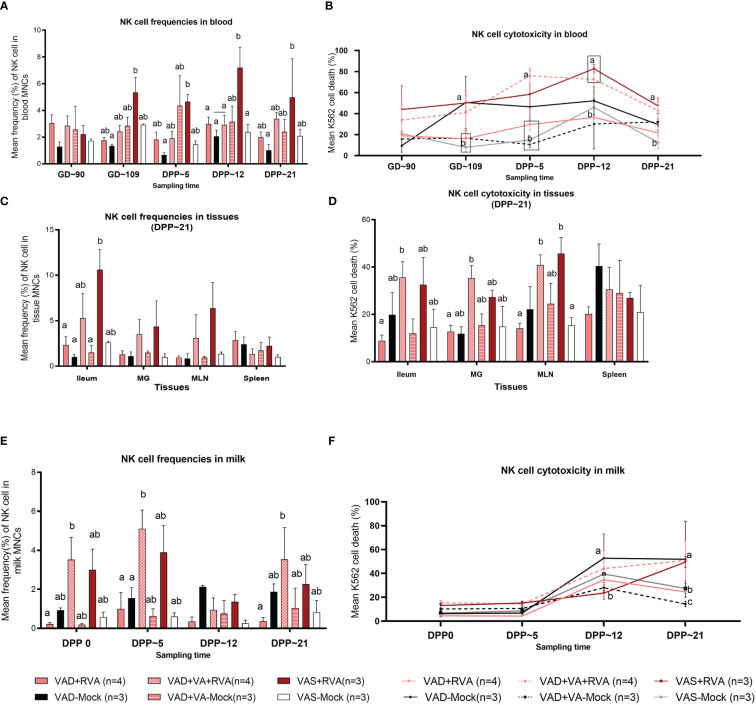
Natural killer (NK) cell frequencies and cytotoxicity analysis by flow cytometry. Sows were fed VAD or VAS diet from GD~30. A subset of VAD sows was given VA supplementation starting at GD~76 to the end of experiment. Blood, milk, and tissues were collected at GD~90 (blood), GD~109 (blood), DPP0 (milk), DPP~5 (milk &blood), DPP~12 (milk &blood) and DPP~21(milk, blood &tissues). Mononuclear cells were isolated and stained with porcine Abs against NK cell markers. **(A)** Mean frequencies of NK cells in blood. **(C)** Mean frequencies of NK cells in tissues **(E)** Mean frequencies of NK cells in milk. K562 cells were stained with CFSE and incubated with isolated mononuclear cells overnight. 7AAD was used to determine K562 cell death (CFSE^+^7AAD^+^) using flow cytometry. **(B)** NK cell activity (K562 cell death %) in blood **(D)** NK cell activity in tissues **(F)** NK cell activity in milk. Letters a and b indicate significant differences among treatment groups (mean ± SEM) at each time point. Statistical analysis was performed using two-way ANOVA with repeated measures and Tukey-Kramer test for multiple comparisons, *P* ≤ 0.05. (GD, gestation day; DPP, day post-partum; VAD, vitamin A deficiency; VAS, Vitamin A sufficient; VA-vitamin A).

NK cell frequencies were decreased in blood of VAD+RVA sows when compared with VAS+RVA sows prior to parturition (GD~109) and throughout lactation until euthanasia (DPP~21), with a significant decrease observed at GD~109 and DPP~21 (**Figure 3A**), suggesting that VAD decreased blood NK cell frequencies during RVA infection. NK cell frequencies were also decreased in ileum, MLN, and MG of VAD+RVA sows when compared to VAS+RVA (**Figure 3C**). Notably, NK cell frequencies were significantly lower in VAD+RVA, VAD-Mock and VAD+VA-Mock in ileum (**Figure 3C**), indicating that VAD decreased NK cell numbers recruited to the RVA infection site (ileum). In milk, we observed a decrease in the frequencies of NK cells in VAD+RVA sows and all mock sows throughout lactation, while VAS+RVA and VAD+VA+RVA sows had comparable elevated frequencies of NK cells in early lactation at DPP0 and DPP~5 (**Figure 3E**), demonstrating that VAD decreased NK cell frequencies in milk and VA supplementation to VAD sows increased these cells in milk. Notably, NK cells in VAS-Mock and VAD-Mock sows were lower in milk throughout the experiment, suggesting that RVA inoculation during gestation resulted in increased NK cell frequencies.

#### 3.3.2 Vitamin A supplementation increased NK cells activity in VAD+RVA sows

To further understand the effects of VAD on the NK cell function, we performed NK cytotoxicity assays using isolated MNCs as effector cells and K562 cells (expressing ligands for NK cells required to induce NK cell death) as target cells (50). In blood MNCs incubated with K562 cells, we observed that the mean cytotoxicity (% dead K562 cells) was significantly decreased in VAD+RVA, VAD+VA-Mock, and VAS-Mock sows compared with VAS+RVA sows pre-parturition (GD~109) (**Figure 3B**), which coincided with low NK cell numbers observed in blood MNCs. However, VAD-Mock sows had significantly (GD~109) and numerically higher (DPP~5 and DPP~12) NK cell activity in blood when compared with VAD+VA-mock, VAD+RVA and VAS-mock (**Figure 3B**), suggesting that VAD might have induced upregulation of pro-inflammatory state through downregulation of MHC I expression in cells. Evaluation of the NK cell activity in selected tissues revealed that NK cell activity was decreased in VAD+RVA in all tissues (**Figure 3D**), suggestive of a combined effect of VAD and RVA infection. Further, we observed significantly (MLN) and numerically (ileum, MG, and spleen) decreased NK cell activity in VAD+RVA, VAD-mock and VAS-mock when compared with VAS+ RVA and VAD+VA+RVA (**Figure 3D**), revealing that VA supplementation to VAD+RVA sows increased NK cell activity in these tissues during RVA infection. Interestingly, splenic NK cell activity of VAD-Mock was higher (but not significantly) than in any other group. Similarly, we observed significantly increased NK cell activity in the milk of VAD-Mock sows compared with VAS+RVA (DPP~12) and VAD+RVA, VAS-Mock and VAD+VA-Mock (DPP~21) sows (**Figure 3F**), further corroborating the fact that VAD dysregulates NK cell normal activity.

### 3.4 Evaluation of frequencies of dendritic cell subpopulations

#### 3.4.1 VAD decreased the frequencies of MHCII^+^ cDCs and pDCs in tissues, blood and milk of VAD ( ± RVA) sows

**Figure 4:**
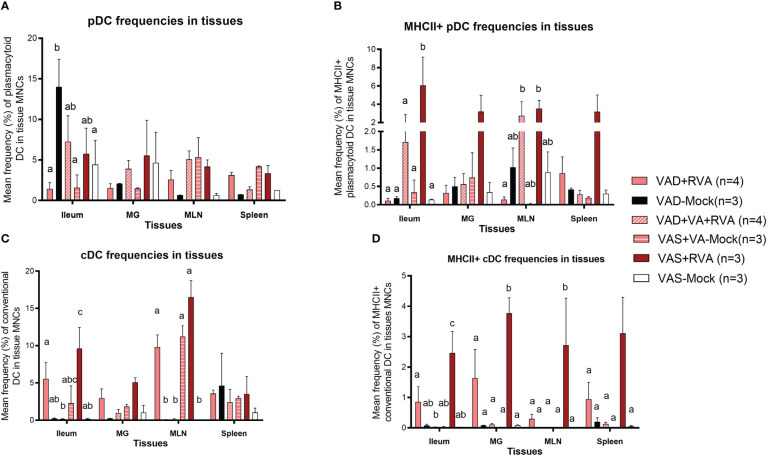
Analysis of different subsets of pDCs and cDCs in local and systemic tissues by flow cytometry. Ileum, MG, MLN, and spleen were collected at ~DPP21 (Euthanasia) and mononuclear cells were isolated. Mononuclear cells were analyzed by flowcytometry. **(A)** Mean frequencies of total plasmacytoid DCs in tissue mononuclear cells. **(B)** Mean frequencies of MHCII^+^ plasmacytoid DCs in tissue mononuclear cells. **(C)** Mean frequencies of total conventional DCs in tissue mononuclear cells. **(D)** Mean frequencies of MHCII^+^ conventional DCs in tissue mononuclear cells. Letters a, b and c indicate significant differences among treatment groups (mean ± SEM) at each time point. Statistical analysis was performed using two-way ANOVA with repeated measures and Tukey-Kramer test for multiple comparisons, *P* ≤ 0.05. (GD, gestation day; DPP, day post-partum; VAD, vitamin A deficiency; VAS, Vitamin A sufficient; VA-vitamin A).

We evaluated the frequencies of different subsets of DCs in the gut mucosa-associated tissues (ileum and MLN), spleen (systemic) and MG due to its role in lactogenic passive immunity. We observed a decrease in the frequencies of total pDCs in VAD ( ± RVA) sows compared with VAS+RVA sows in MLN, MG, spleen, and ileum (only VAD+RVA) (**Figure 4A**). VA supplementation to VAD+RVA sows (VAD+VA+RVA) increased total pDCs frequencies in all the tissues except in MLN and spleen. Unexpectedly, VAD-mock sows had significantly higher frequencies of total pDCs in ileum compared with VAD+RVA, VAD+VA-Mock and VAS-Mock; however, further evaluation revealed low expression of MHCII in these pDCs (**Figure 4B**), corroborating our earlier results that VAD causes pDCs impairment. In addition, analysis of MHCII^+^ pDCs showed significantly (Ileum, MLN) and numerically (MG and spleen) higher frequencies of these cells in VAS+RVA than in VAD+RVA (**Figure 4B**). Moreover, VA supplementation increased MHCII^+^ pDCs frequencies in ileum, MG, and MLN of VAD+RVA sows. Furthermore, we observed numerically (VAD+RVA) and significantly (VAD-Mock) lower frequencies of total and MHCII^+^ cDCs in ileum and MLN compared to VAS+RVA sows (**Figures 4C, D**). Unexpectedly, supplementing VA to VAD sows (VAD+VA ± RVA) did not increase MHCII^+^ cDCs frequencies in any of the tissues analyzed (**Figure 4D**). Overall, these results reveal that VAD decreased total and MHCII^+^ pDCs and cDCs frequencies in the blood, ileum, MLN, MG and spleen and that VA supplementation increased some but not all decreased frequencies of DCs.

The frequencies of total pDCs in blood were numerically decreased in blood in VAD ( ± RVA) sows at all sampling points, except at DPP~21 where VAD-Mock had numerically higher frequencies of these cells than all other groups (**Figure S2A**). There was a steady increase in the frequencies of pDCs and MHCII^+^pDCs in blood of VAD+VA+RVA sows except at DPP~21 indicating that VA supplementation increased pDC frequencies in blood over time (**Figure S2A, B**). Similarly, we observed a decrease in total and MHCII^+^cDC frequencies in VAD+RVA than VAS+RVA sows throughout the experiment except at DPP~21 (total cDCs) (**Figure S2C, D**). Interestingly, total and MHCII^+^cDC frequencies were increased in VAD-Mock in mid to late lactation, suggesting enhanced VA mobilization from the liver reserves by these sows or dysregulation due to VAD. Further, total and MHCII^+^cDC frequencies were gradually increased in VAD+VA+RVA sows pre- and post-partum implying that VA supplementation restored total cDC frequencies in blood (**Figure S2D**), further accentuating the importance of VA in regulating DCs numbers.

Analysis of DC frequencies in milk revealed a significant decrease in total cDCs frequencies in VAD ( ± RVA) and in mock animals regardless of their diet when compared with VAS+RVA and VAD+VA+RVA sows at DPP0 and DPP~5 (**Figure S3A**). Further evaluation of MHCII^+^ cDCs revealed a significant increase in colostrum (DPP0) of VAD+VA+RVA when compared with VAD+RVA and VAS-Mock sows (**Figure S3B**), suggesting that VA supplementation and RVA inoculation increased the expression of MHCII^+^ in cDCs.

#### 3.4.2 Vitamin A deficiency decreased the frequencies CD103^+^ DCs in blood, ileum, MLN, and MG

**Figure 5:**
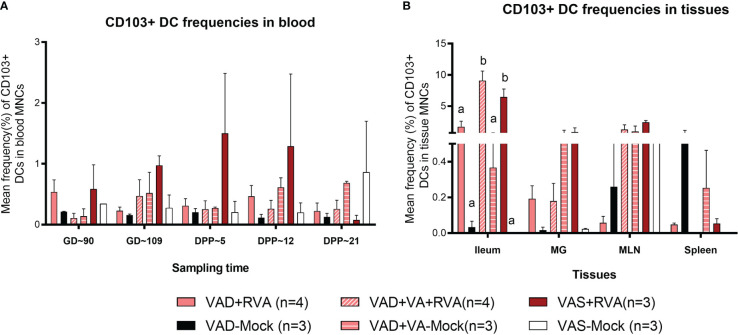
Analysis of CD103^+^ DCs in blood and tissues using flow cytometry. Mean frequencies of CD103^+^ dendritic [CD103^+^ DCs] among blood MNCs **(A)** and tissues **(B)**. Letters a and b indicate significant differences among treatment groups (mean ± SEM) at each time point. Statistical analysis was performed using two-way ANOVA with repeated measures and Tukey-Kramer test for multiple comparisons, *P* ≤ 0.05. (GD, gestation day; DPP, day post-partum; VAD, vitamin A deficiency; VAS, Vitamin A sufficient; VA-vitamin A).

We evaluated the effects of VAD on CD103^+^ DCs due to their role in VA metabolism and tolerance induction to self, food antigens and beneficial microbes in the gut. Overall, we observed a gradual increase in CD103^+^ DCs frequencies in blood of VAS+RVA during gestation to mid-lactation before sharply decreasing in late lactation (DPP~21) (**Figure 5A**). The frequencies of CD103^+^ DCs in blood of VAD ( ± RVA) sows were decreased throughout the experiment and VA supplementation did not increase the frequency of these cells in blood (**Figure 5A**). In the tissues evaluated, CD103^+^ DCs frequencies were significantly lower in ileum, and numerically decreased in MG and MLN of VAD+RVA sows compared with VAS+RVA and VAD+VA+RVA (ileum) sows (**Figure 5B**), suggesting that VAD impacted CD103^+^ DC frequencies at the site of infection. In addition, CD103^+^ DCs frequencies were significantly lower in the ileum of VAS-mock and VAD -mock ( ± VA) compared with VAS+RVA and VAD+VA+RVA respectively (**Figure 5B**), suggesting an effect of anamnestic (secondary) immune responses in the RVA immunized animals. Moreover, VA supplementation of VAD sows increased the frequency of CD103^+^ DCs in both ileum and MLN. Taken together, these results suggest that VAD causes an imbalance in CD103^+^ DCs frequencies in both systemic and the gut-associated tissues.

### 3.5 Vitamin A deficiency decreased the frequencies of CD4^+^ and CD8^+^ T-regulatory and RVA-specific IFN-γ producing T cells

**Figure 6:**
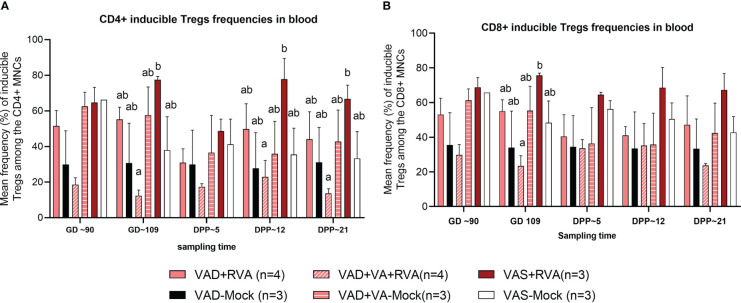
Analysis of CD4^+^ and CD8^+^ inducible regulatory T cells in blood using flow cytometry. **(A)** Mean frequencies of CD4^+^ inducible among the CD4^+^ T cells in blood mononuclear cells. **(B)** Mean frequencies of CD8^+^ inducible among the CD8^+^ T cells in blood mononuclear cells. Letters a and b indicate significant differences among treatment groups (mean ± SEM) at each time point. Statistical analysis was performed using two-way ANOVA with repeated measures and Tukey-Kramer test for multiple comparisons, *P* ≤ 0.05. (GD, gestation day; DPP, day post-partum; VAD, vitamin A deficiency; VAS, Vitamin A sufficient; VA-vitamin A).

There were no clear trends in the frequencies of natural and activated Tregs (data not shown); however, there were differences in the frequencies of induced Treg cells (iTregs) among the groups. We observed decreased frequency of CD4^+^ iTregs in blood of VAD ( ± RVA) sows compared to VAS+RVA sows throughout the experiment (**Figure 6A**). Interestingly, there were a significantly (GD109, DPP~5 and DPP ~21) and numerically (GD~90, DPP~12) lower CD4^+^ iTreg frequencies in VAD+VA+RVA sows when compared with VAS+RVA sows (**Figure 6A**), suggesting that VA supplementation to VAD+RVA sows did not increase frequencies of CD4^+^ iTregs in blood. Moreover, we observed similar trends in frequencies of CD8^+^ inducible Tregs in blood (**Figure 6B**).

**Figure 7:**
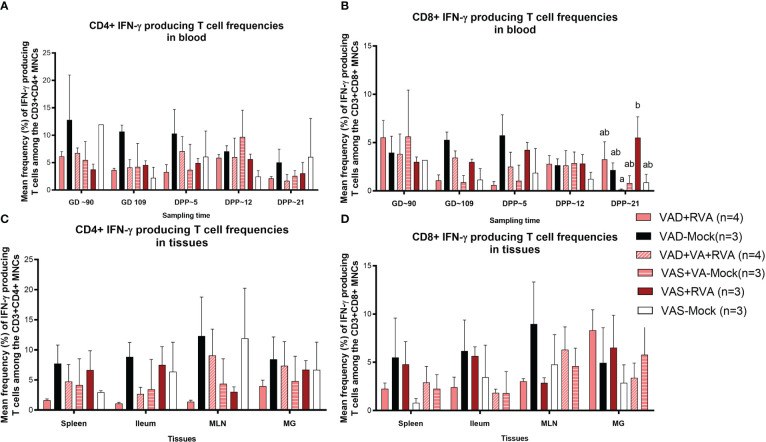
Analysis of RVA-specific IFN-γ secreting T cells by flow cytometry. Mononuclear cells were restimulated with RVA virus *in vitro.* **(A)** Mean frequencies of RVA-specific IFN-γ producing CD4^+^ T cells among CD3^+^CD4^+^ T cells in blood mononuclear cells. **(B)** Mean frequencies of RVA-specific IFN-γ producing CD8^+^ T cells among CD3^+^CD8^+^ T cells in blood mononuclear cells. **(C)** Mean frequencies of RVA-specific IFN-γ producing CD4^+^ T cells among CD3^+^CD4^+^ T cells in tissue mononuclear cells **(D)** Mean frequencies of RVA-specific IFN-γ producing CD8^+^ T cells among CD3^+^CD8^+^ T cells in tissue mononuclear cells. Letters a and b indicate significant differences among treatment groups (mean ± SEM) at each time point. Statistical analysis was performed using two-way ANOVA with repeated measures and Tukey-Kramer test for multiple comparisons, *P* ≤ 0.05. (GD, gestation day; DPP, day post-partum; VAD, vitamin A deficiency; VAS, Vitamin A sufficient; VA-vitamin A).

We also evaluated RVA-specific IFN-γ secreting T cell subpopulations in blood, milk, and tissues. IFN-γ is a cytokine produced by cells of the innate immune system and T cells and is responsible for increasing MHCII expression and antigen presentation in DCs. We observed an increase in RVA-specific IFN-γ producing CD4^+^ (GD~90, GD~109 and DPP~5) and CD8^+^ (GD~109 and DPP~5) T cell frequencies in blood of VAD-mock sows, in which RVA specific IFN-γ producing CD4^+^ and CD8^+^ T cells steadily decreased from DPP~5 to DPP21 (**Figures 7A, B**). Interestingly, RVA-specific IFN-γ producing CD8^+^ T cell frequencies were significantly (VAD+VA+RVA) and numerically (VAD+RVA, VAD-Mock, VAS-mock) decreased compared with the VAS+RVA sows at DPP~21 (**Figure 7B**). Additionally, we observed higher RVA-specific IFN-γ producing CD4^+^ T cell frequencies in all tissues evaluated of VAD-Mock sows (except for MLNs where VAS-Mock sows had higher RVA-specific IFN-γ producing CD4^+^ T cell frequencies) than in all other groups (**Figure 7C**). A similar trend was observed in ileum, MLN, and spleen for RVA-specific IFN-γ producing CD8^+^ T cell frequencies (**Figure 7D**). Taken together, our results suggest that VAD decreased RVA-specific IFN-γ producing T cells while VAD alone increased these cells.

### 3.6 Vitamin A sufficient and RVA inoculated sows had decreased IL-10 and IL-22 in sow serum

**Figure 8:**
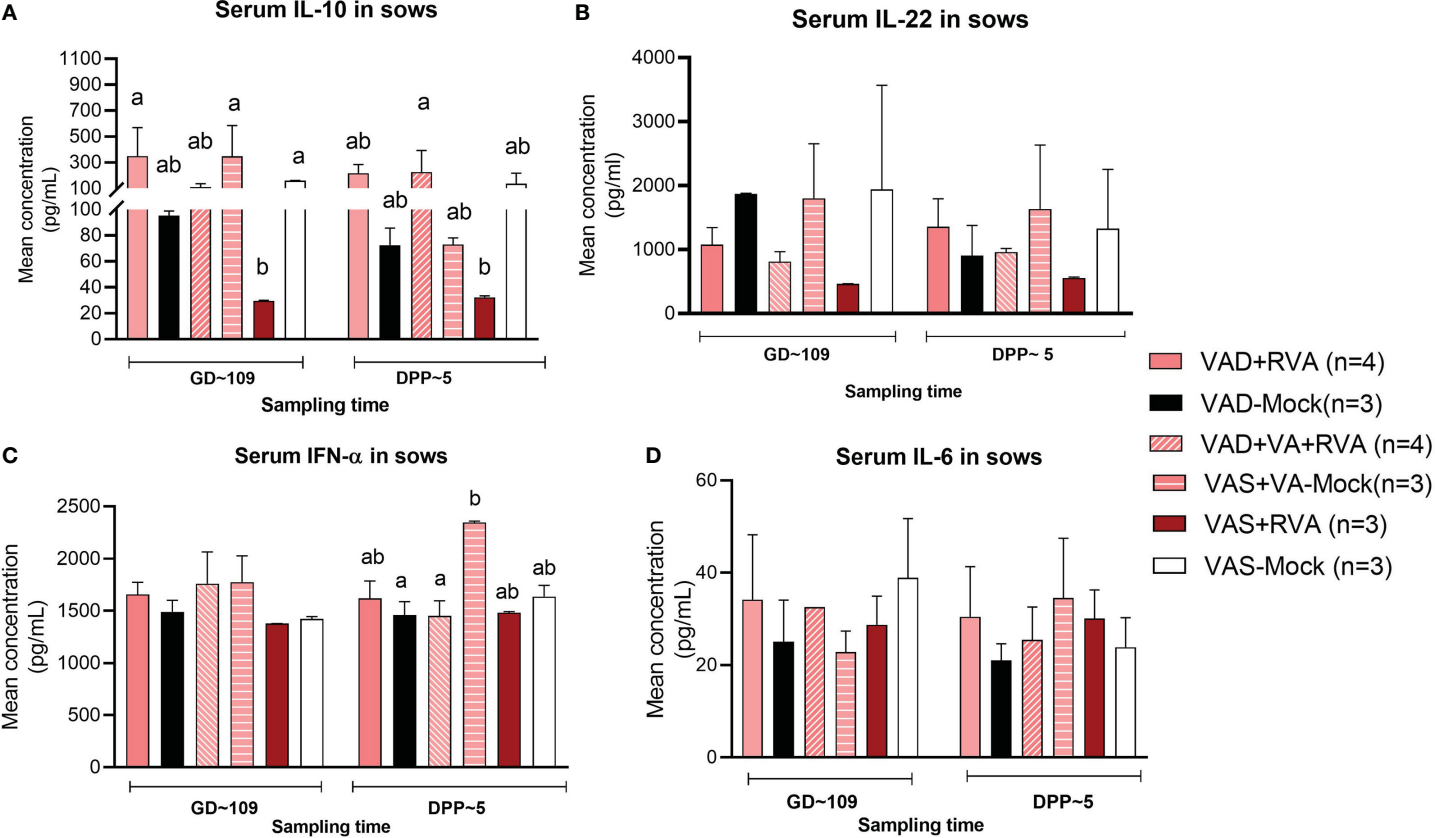
Pro-inflammatory and anti-inflammatory cytokine concentration in blood of sows. Blood was collected from sows at GD~109 (pre-partum) and DPP~5 (pre-partum) for cytokine analysis. **(A)** Mean concentration of serum IL-10. **(B)** Mean concentration of serum IL-22. **(C)** Mean concentration of serum INF-α. **(D)** Mean concentration of serum IL-6. Letters a and b indicate significant differences among treatment groups (mean ± SEM) at each time point determined using Kruskal-Wallis test and Dunn’s multiple comparison test, *P* ≤ 0.05. (GD, gestation day; DPP, day post-partum; VAD, vitamin A deficiency; VAS, Vitamin A sufficient; VA-vitamin A).

Cytokines play a key role in both innate and adaptive immune responses and their role is critical in the timely clearing of infection. Thus, we evaluated the effects of VA status and RVA inoculation on production of selected innate, pro- and anti-inflammatory cytokines (IL-6, IL-10, IL-22 and IFN- α). Our results showed that both IL-10 (anti-inflammatory) and IL-22 (pro-/anti-inflammatory) levels in serum were significantly (IL10 at GD~109) and numerically decreased in VAS+RVA sows compared with other groups pre- and post-partum (**Figures 8A, B**). Moreover, IL-10 levels were decreased post-partum in VAD+VA-Mock sows, while they were increased in VAD+VA+RVA (**Figure 8A**). On the other hand, the levels of IFN-α (innate, pro- -inflammatory) and IL-6 (pro-inflammatory) were similar pre- and post-partum in all groups, although VAD+VA-Mock animals had increased IFN-α (significantly) and IL-6 (relatively) post-partum and vice versa, IL-6 was decreased in VAS-mock post-partum (**Figures 8C, D**).

### 3.7 Vitamin A deficiency decreased the expression of pIgR and RARα in Ileum and MLNs

We evaluated the gene expression of pIgR by RT-qPCR in mucosal tissues due to its role in facilitating transcytosis of the secreted dimeric IgA. We observed decreased expression of pIgR in MLNs and ileum of VAD sows, with the largest decrease observed in ileum (**Table 2**). VA supplementation to VAD sows increased pIgR expression in both ileum and MLN of these animals.

**Table 2:** Analysis of sow ileal and MLN mRNA levels of different genes by RT-qPCR relative to VAS sows at DPP~21(mean ± SEM).

	Ileum		MLN	
Gene	VAD	VAD+VA	VAD	VAD+VA
pIgR	0.18 ( ± 0.07)	0.97 ( ± 0.7)	0.84 ( ± 0.25)	0.82 ( ± 0.61)
RARα	0.59 ( ± 0.20)	0.93 ( ± 0.3)	0.74 ( ± 0.22)	1.30 ( ± 0.18)
RPB4	0.93 ( ± 0.1)	0.98 ( ± 0.07)	0.89 ( ± 0.1)	0.79 ( ± 0.3)
MAdCAM-1	1.73 ( ± 0.2)	1.36 ( ± 0.4)	1.42 ( ± 0.4)	1.57 ( ± 0.9)
VCAM-1	1.16 ( ± 0.25)	0.53 ( ± 0.17)	0.60 ( ± 0.1)	0.35 ( ± 0.1)

To understand the association between VA status, and transportation of VA in sows during and post RVA inoculation, we evaluated the expression of retinol binding protein 4 (RBP4, protein that transports retinal to the tissues) and RA receptor (RARα, RA receptor in the cells) genes in ileum and MLN. All groups had comparable expression levels of RBP4, while RARα expression was low in both ileum and MLN of VAD+RVA sows (**Table 2**). However, VA supplemented VAD sows (VAD+VA) had increased RARα mRNA expression. Taken together, our results suggest that VAD impairs pIgR and RARα gene expression but not RBP4 gene expression in the gut.

Additionally, we evaluated the expression of the cellular adhesion molecules, VCAM-1 and MAdCAM-1 by RT-qPCR due to their roles in mediating lymphocyte migration to certain systemic and mucosal tissues, respectively. Surprisingly, MAdCAM-1 expression was increased in VAD and VAD+VA sows in Ileum and MLN suggestive of a possible VA compensatory mechanism. However, VCAM-1 expression was increased in ileum, but decreased in MLN in VAD sows (**Table 2**). VA supplemented VAD sows maintained low VCAM-1 expression (**Table 2**). Thus, the effect of VAD on MadCAM-1 and VCAM-1 in sows is not clear and requires further investigation.

## 4 Discussion

Vitamin A deficiency is one of the most serious global public health concerns, especially in developing countries, leading to different subclinical, clinical symptoms and even mortality in children (51). Moreover, VAD contributes to the low efficacy of vaccines against enteric diseases such as polio vaccine in infants and pre- school children in LMIC (52). Overall, the efficacy of most vaccines in the developing world is low and partially attributed to the high prevalence of VAD in young children and pregnant/lactating mothers in LMIC (12, 35, 53, 54). Many clinical and animal studies on RVA have focused on immune responses of neonates and young children; however, the role of VAD and maternal RVA immunization during gestation on maternal immune responses and passive protection of their offspring is not well understood. We recently demonstrated that VAD impairs B cell immune responses in pregnant and lactating sows, lowering passive protection of their suckling piglets against RVA (41). Therefore, there is a need to determine if VAD also impairs innate and T cell immune responses largely responsible for balanced immune responses against infection.

The contribution of VAD to immune impairment has been evaluated (demonstrated) in different animal models. Bowman and colleagues showed that NK cell activity was decreased in VAD rats (55), similar to our observation in conventional sows. Zhao and coauthors, using a rat model, revealed that VAD did not affect NK cell activity in blood MNCs, but decreased NK frequency and activity in the spleen (22). In this study we observed decreased NK cell frequency and activity in blood, milk, ileum, MG, and MLN MNCs but not spleen in VAD (± RVA) sows. The difference might be due to RVA infection in our study affecting NK cells frequencies and activity mainly in the local mucosal tissues and/or differences in swine and rodent immune responses (56). Studies using a mouse model revealed that dietary and pharmacologically induced VAD depleted splenic RA-dependent DCs suggesting that VA played a key role in DC maturation and maintenance (57). Similarly, we observed that VAD( ± RVA) decreased both pDC and cDC frequencies and expression of MHCII on these cells in pregnant and lactating sows. In contrast, other studies of VAD animal models have shown that VAD induces increased systemic and mucosal DCs, indicative of skewed immune responses rather than increased DC activity (35, 58). We observed increased total and MHCII^+^ cDC, but not pDC frequencies in assessed tissues of VA supplemented VAD+RVA sows, similar to our previous observations in Gn piglets, where VA supplementation failed to restore all prenatally induced VAD immune impairment (35).

Our lab has previously shown that VAD decreased CD103^+^ DCs in Gn piglets (35), which was further corroborated by our current study. Furthermore, we have shown that expression of gut homing receptors α4β7/CCR10 on blood B cells was decreased in VAD+RVA sows (41), suggesting that the decreased CD103^+^ DCs affected expression of these markers on B cells. VA supplementation of RVA infected VAD sows (VAD+VA+RVA) increased the frequency of CD103^+^ DCs in blood prepartum (GD~109), MLN and ileum, suggesting that VA is also required for maintaining adequate levels of these cells in the gut. Overall, these results suggest that VAD alters the numbers of both pDCs and cDCs and their subsets in pregnant and lactating sows. Because DCs bridge innate and adaptive immunity, this explains the impaired adaptive (B cell) immune response that we observed in our prior study of the same group of animals (41). Notably, the highest frequency of total cDCs was observed in MLNs indicating that VA might play a greater role in increasing these cell frequencies in the MLNs compared to other assessed tissues, since MLN are the draining lymph nodes for the gastrointestinal tract involved in lymphocyte activation during RVA infection (59).

Soerens and colleagues, using a mouse model of herpes virus, showed that Tregs are critical for homing of DCs from the vaginal mucosa to the draining lymph nodes, leading to effective CD4^+^ T-cell priming, activation, and trafficking to the infected tissues (60). In accordance with the decreased frequencies of MHCII^+^ DCs, we observed a decrease in CD4^+^ inducible Tregs in VAD+RVA sows. There is a complex inter-relationship between DCs and T cells regulated by VA levels; however, the precise mechanisms remain unclear. Whether altered DC numbers and function in VAD led to the altered T cell frequencies observed is an area for further investigation. Unexpectedly, VA supplementation did not restore the reduced numbers of inducible Tregs in the VAD+RVA infected sows.

Plasmacytoid DCs activate T cells to produce IFN-γ *via* antigen presentation to naïve T cells enhancing their antiviral activity and effector properties (61). We observed higher frequencies of RVA-specific IFN-γ producing CD4^+^ and CD8^+^ T cells in blood, spleen, ileum, MLN and MG of VAD mock (RV seropositive, non-RVA inoculated) sows after MNCs *in vitro* RVA stimulation, which is in agreement with previous findings showing that VAD favors Th1 over Th2 responses in mice (62, 63). Interestingly, VAD+RVA sows had lower RVA-specific IFN-γ CD4^+^/CD8^+^ than VAD-mock sows suggesting that RVA inoculation in VAD sows decreased Th1 bias during restimulation. VAS+RVA sows had lower IL-10 and IL-22 compared to all other groups. Studies have shown that some pathogens can harness the immunosuppressive capacity of IL-10 to limit host immune responses, leading to persistent infection and increase in IFN-γ producing T cells (64, 65). Moreover, our observation is consistent with Yang et al. where VAD mice had increased IL-10 levels compared to VAS mice (66). Downregulation of Th2 responses might have contributed to decreased RVA specific IgA and IgG ASCs, IgA^+^ B cells and CCR10^+^ B cells in VAD+RVA sows observed in our recent study, leading to decreased RVA specific IgA and IgG Abs in serum and intestinal contents of VAD+RVA litters (41). Furthermore, the increased RVA RNA shedding, and diarrhea frequency observed in VAD-mock litters suggest a role of VA and RVA in induction/maintenance of adequate passive protection. Moreover, we have previously shown that pro-inflammatory IFN-α was increased in VAD Gn pigs (35); likewise our current study of conventional pregnant and lactating sows showed that IFN-α and IL-6 were slightly increased in VAD+RVA sows compared with VAS+RVA sows, suggesting that VAD might contribute to hyperinflammation. Our findings are in agreement with Ahmed and colleagues who showed that RA inhibits IL-6 production in human osteoblasts cells in a dose dependent manner (67). Because our sows had subclinical VAD, we hypothesize that IL-6 variation might be more pronounced with further depletion of hepatic VA reserves in VAD sows.

Vitamin A deficiency affects pIgR and RARα expression levels *in vivo* and *in vitro* (68–70). Takenouchi-ohkubo and coworkers showed that RA treatment increased pIgR levels in HT-29 and Caco-2 human intestinal cell lines (70), similar to our observation in a conventional sow model, where VAD sows had decreased pIgR mRNA levels in both ileum and MLN compared with VAS and VAD+VA sows. Decreased pIgR in MLNs and ileum coincided with our earlier observation where RVA specific-IgA Ab levels in large intestinal contents were decreased (41), suggesting that VA is important for pIgR expression and its role in transporting sIgA to the apical side of the epithelial cells. RBP4 is highly expressed in liver followed by adipose and other tissues (71, 72); thus, we observed minimal differences in RBP4 expression levels in ileum and MLN of all sow groups, consistent with observations by Soprano and colleagues in VAD rats (73). However, expression of RARα was decreased in ileum and MLN of VAD sows, suggesting that VA status and infection plays a role in RARα expression and further explaining the impaired innate and adaptive immune responses observed in this study and our previous study (41). Previously, we observed a significant decrease of α4β7 expressing blood B cell frequencies in late gestation (GD~109) and early lactation (DPP~5) in VAD+RVA sows (41). However, in this study, we observed a slight increase in MAdCAM-1, an α4β7 ligand at late lactation (DPP~21), in ileum and MLN of VAD+RVA sows suggesting VAD might not affect expression MADCAM-1 but decreases its receptor α4β7 on lymphocytes affecting early trafficking of these cells.

In conclusion, our study revealed that VAD impairs innate and T cell immune responses, consequently compromising B cell responses and passive protection to suckling piglets. VA supplementation restored some but not all impaired immune responses. Therefore, we conclude that maternal VA supplementation might be necessary in VAD mothers during pregnancy and lactation rather than waiting to supplement their offspring after birth when detrimental damage to the mucosal immune system already occurred and is harder to restore. To our knowledge, this is the first study to investigate the role of VAD and RVA infection (representing anamnestic responses) on innate and T cell immune responses in a conventional sow model. Our results are applicable to the development of optimized RVA vaccines (especially maternal) and VA maternal supplementation for swine and people living in LMIC where VAD rates are high.

## Data availability statement

The original contributions presented in the study are included in the article/**Supplementary Material**. Further inquiries can be directed to the corresponding authors.

## Ethics statement

All animal experiments were approved by the Institutional Animal Care and Use Committee at The Ohio State University under the IACUC.

## Author contributions

LS and AV conceptualized and designed the study and analyzed the data; JC, JA, HM, and KJ conducted the experiments, analyzed the data, and wrote the manuscript; ML helped with animal work and sample processing; AM, SR, DD, and ML assisted with animal work, collected, and processed samples and conducted laboratory experiments; KJ conducted IHC experiments and analysis; LS and AV supervised the work and reviewed and revised the manuscript. All authors contributed to the article and approved the submitted version.
