# Supplementary material for: Vitamin A deficiency and vitamin A supplementation affect innate and T cell immune responses to rotavirus A infection in a conventional sow model
Source: Front Immunol. 2023 Apr 25;14:1188757. doi: 10.3389/fimmu.2023.1188757 (PMC10166828; doi:10.3389/fimmu.2023.1188757)
Supplement: Supplementary file 1 [file DataSheet_1.docx]

**Vitamin A deficiency and vitamin A supplementation affect innate and T cell immune responses to rotavirus A infection in a conventional sow model**

Juliet Chepngeno^1,2^, Joshua O. Amimo^1,3^, Husheem Michael^1^, Sergei A. Raev^1^, Kwonil Jung^1^, Debasu Damtie^4,5^, Alfred Omwando^6^, Anastasia N. Vlasova^1,2*^, Linda J. Saif ^1,2*^

NK cell frequencies


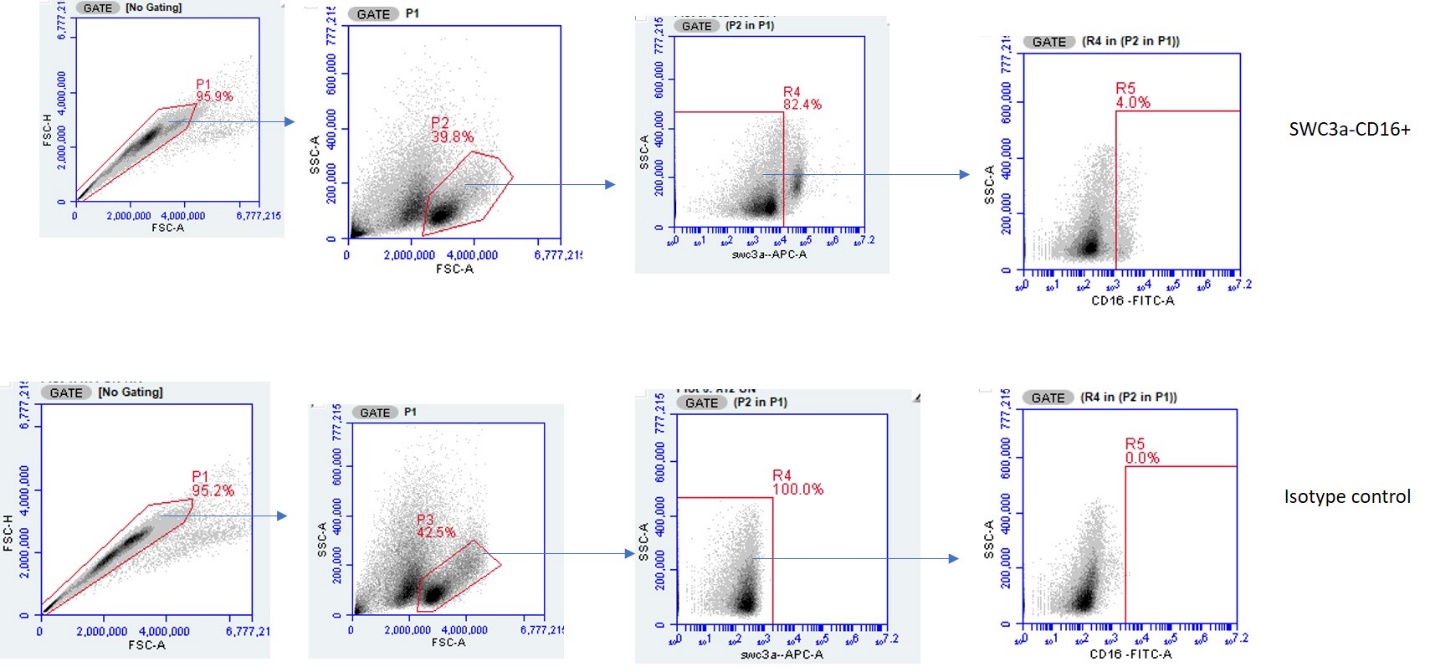


NK cytotoxicity


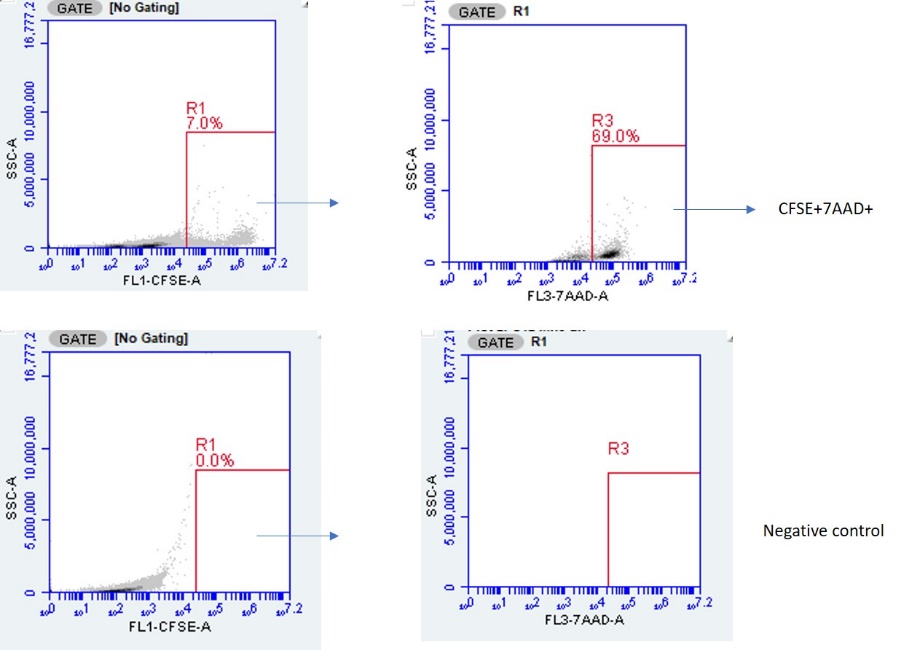


Conventional and plasmacytoid dendritic cell frequencies


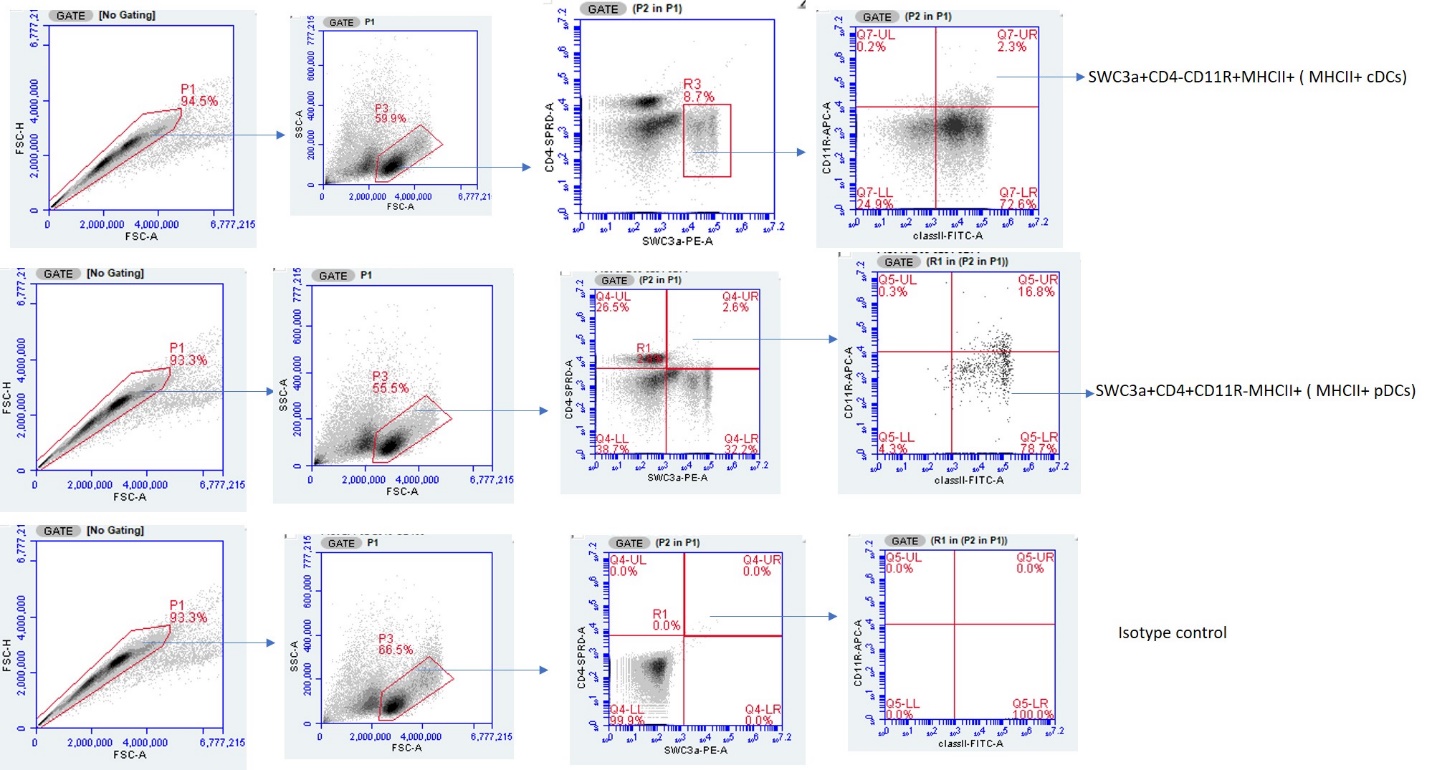


CD103^+^ dendritic cell frequencies

**
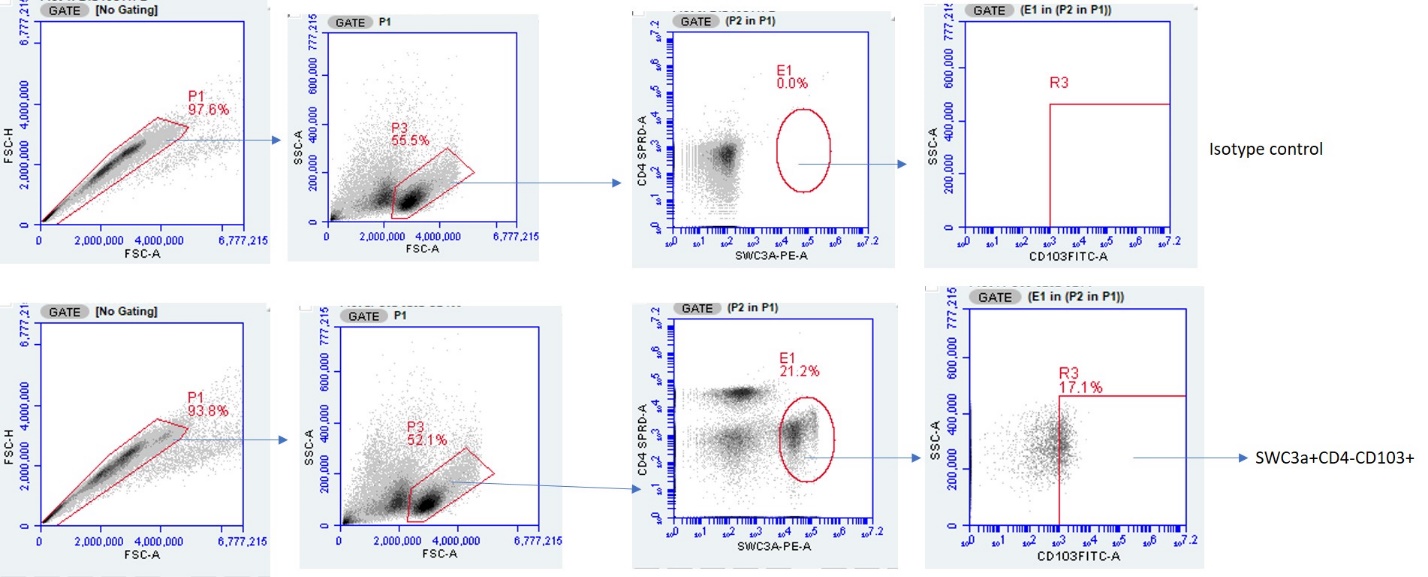
**

T regulatory cell frequencies

**
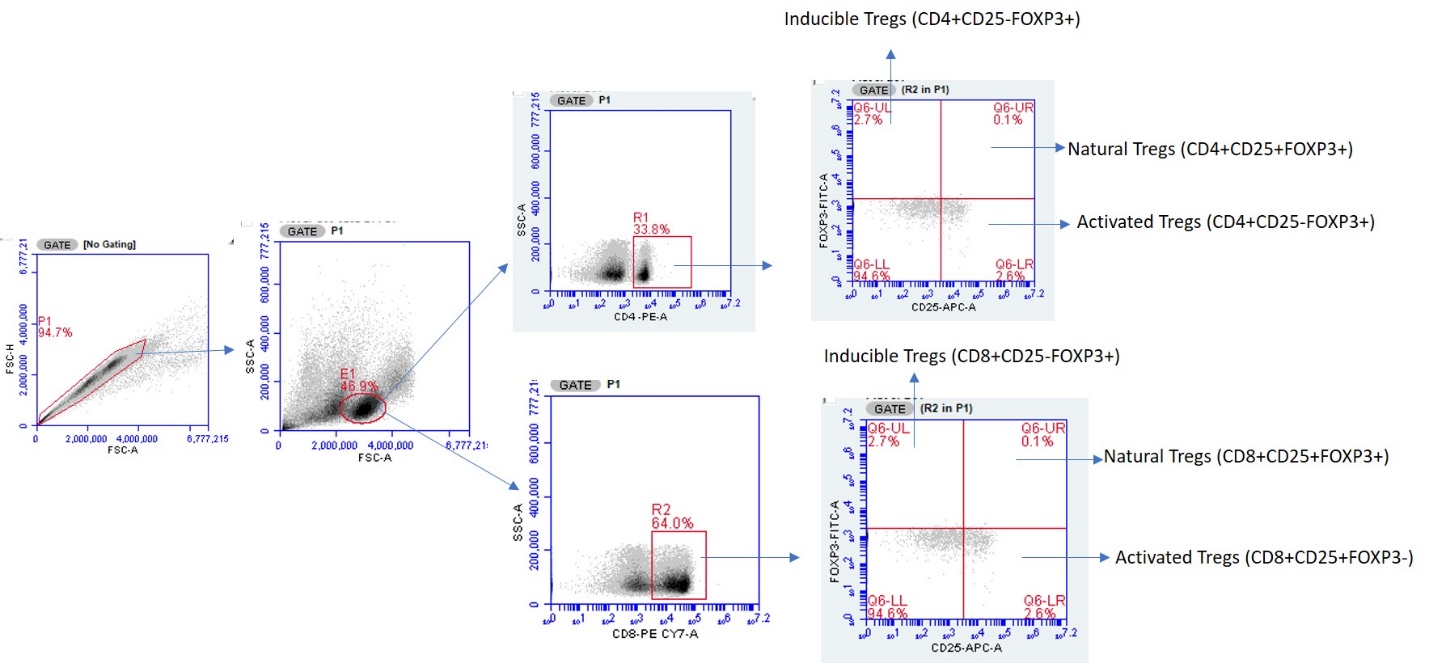
**


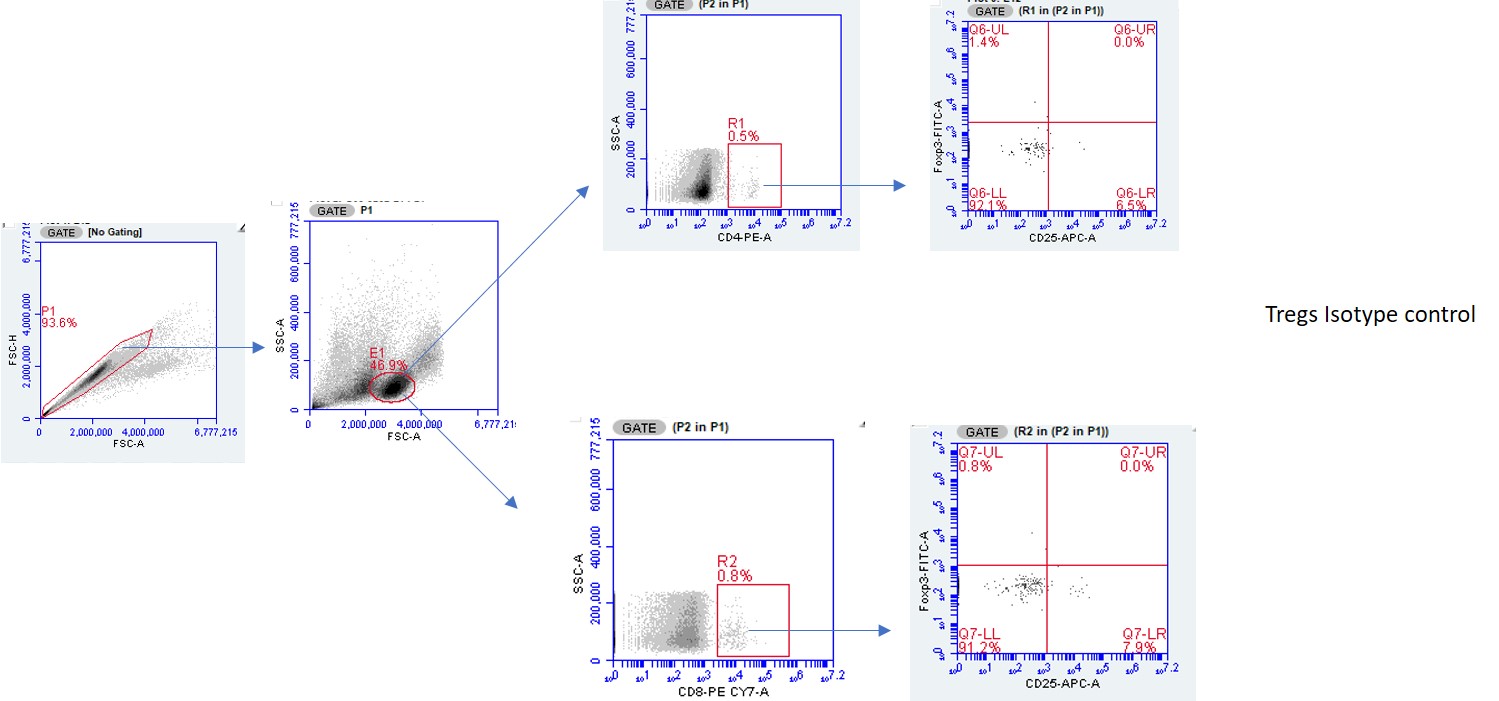


INF-ϒ producing T cells


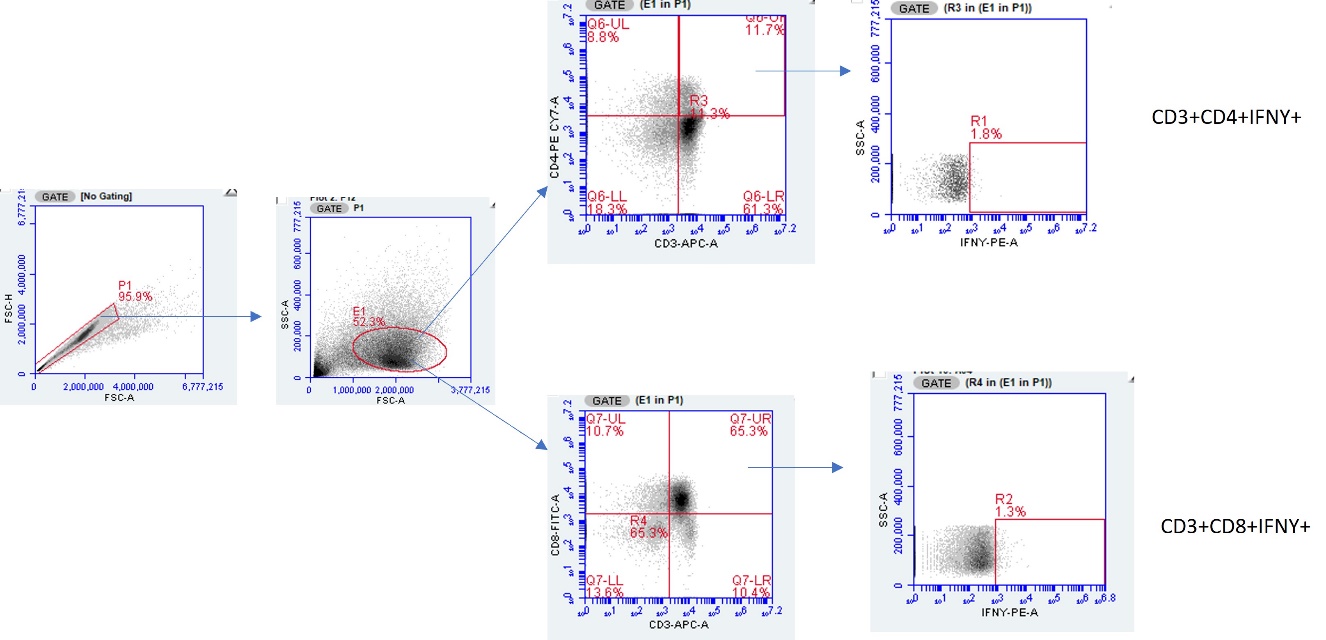


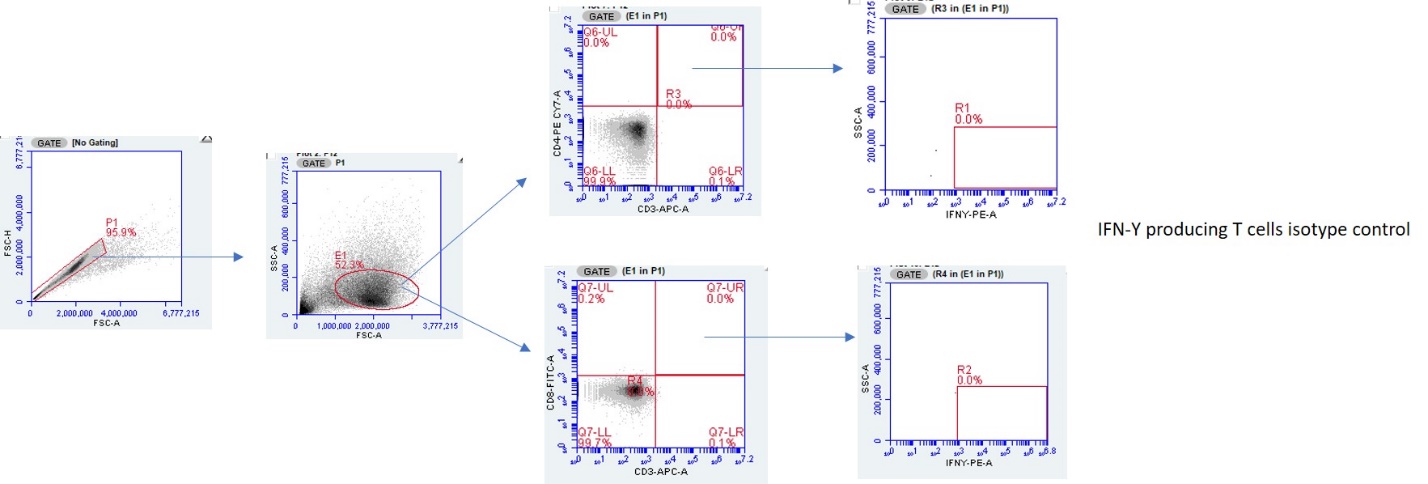


**Figure S1.** Flow cytometry gating strategies for NK cells, NK cell cytotoxicity, plasmacytoid dendritic cells, conventional dendritic cells, T regulatory cells and IFN-ϒ producing T cells.


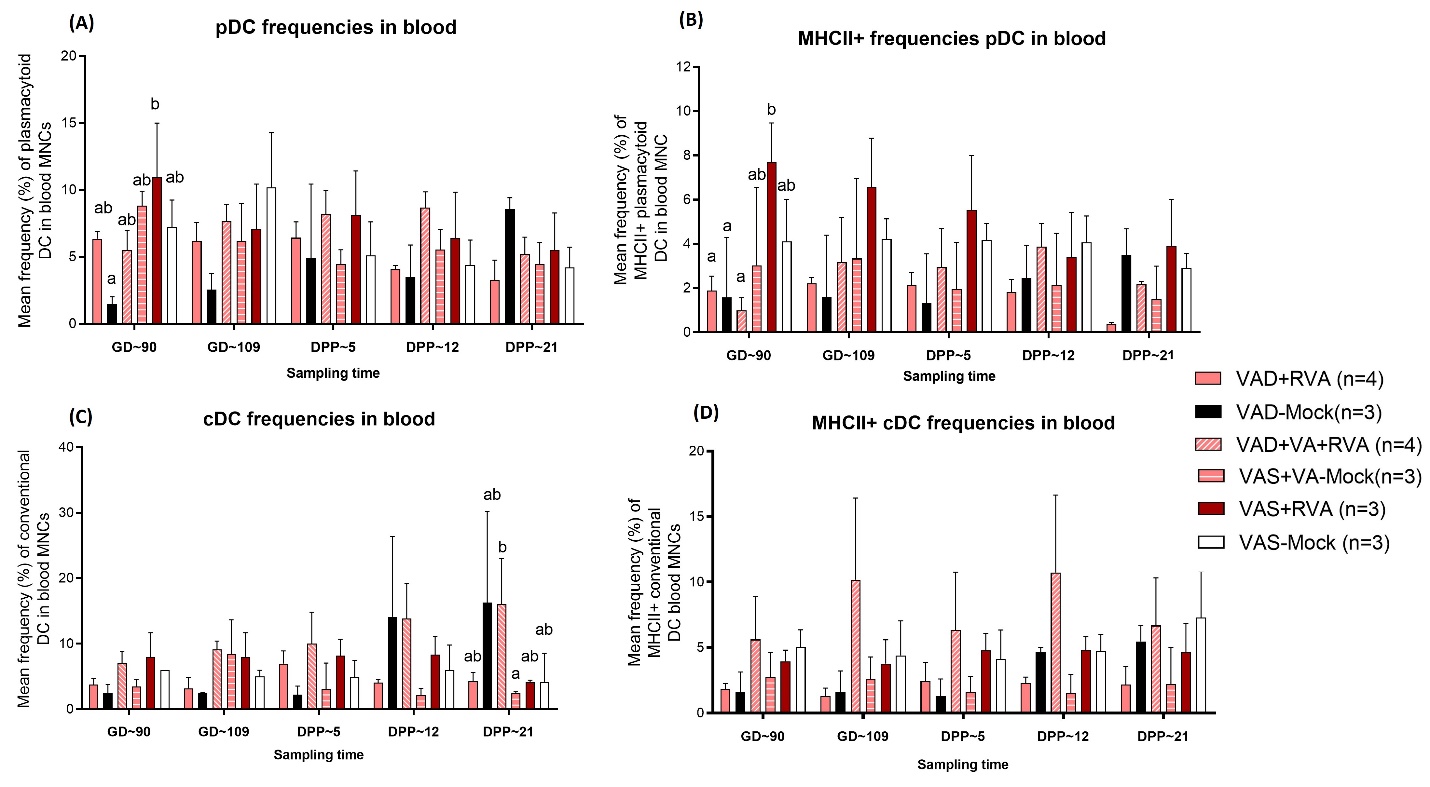


**Figure S2**: **Analysis of different subsets of pDCs and cDCs in blood by flow cytometry**. (**A)** Mean frequencies of total plasmacytoid DCs in blood MNCs. (**B)** Mean frequencies of MHCII^+^ plasmacytoid DCs in blood MNCs. (**C)** Mean frequencies of total conventional DCs in blood MNCs. (**D)** Mean frequencies of MHCII^+^ conventional DCs in blood MNCs. Asterisks indicate significant differences among treatment groups (mean ± SEM). Statistical analysis was performed using two-way ANOVA with repeated measures and Tukey-Kramer test for multiple comparisons. ^*^*P* < 0.05, ^**^*P* < 0.01. (GD=gestation day, DPP=day post-partum, VAD=vitamin A deficiency, VAS=Vitamin A sufficient, VA-vitamin A)


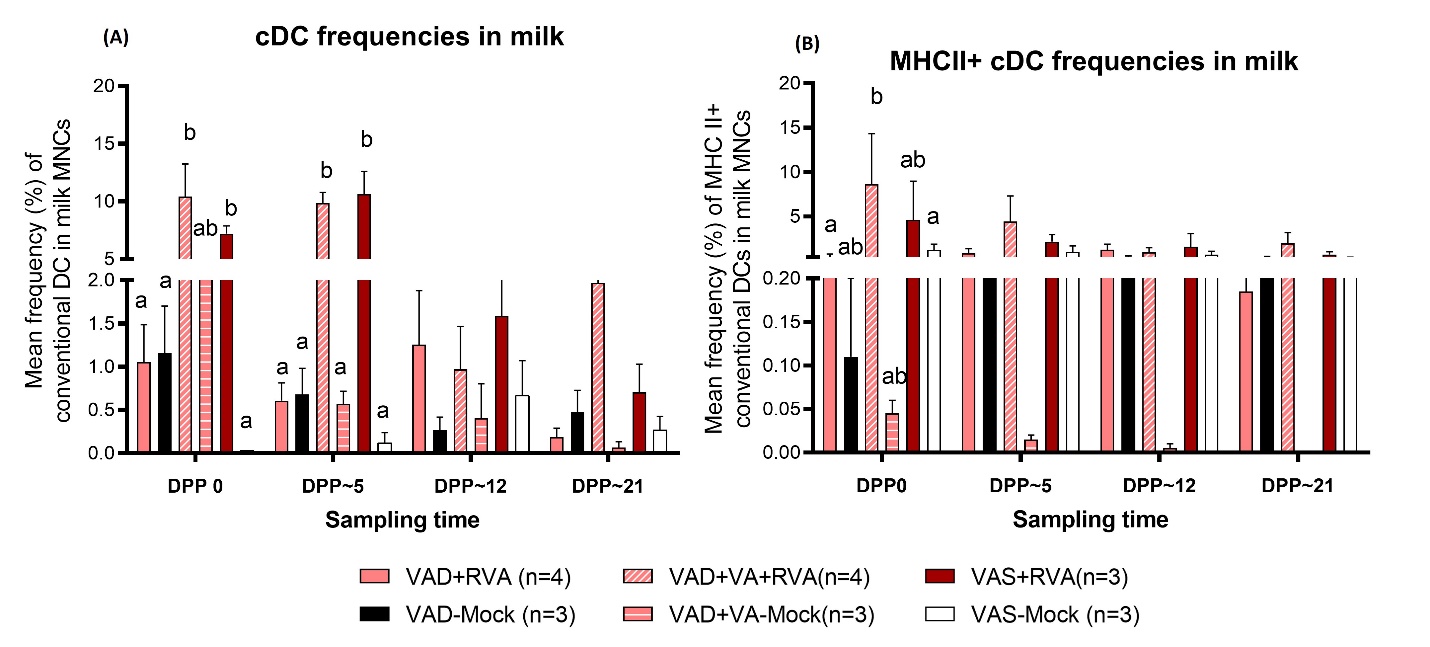


**Figure S3. Analysis of different conventional DC subset frequencies in milk by flow cytometry.** (**A)** Mean frequencies of total conventional DCs in milk MNCs. (**B)** Mean frequencies of MHCII^+^ conventional DCs in milk MNCs. Asterisks indicate significant differences among treatment groups (mean ± SEM). Statistical analysis was performed using two-way ANOVA with repeated measures and Tukey-Kramer test for multiple comparisons. ^*^*P* < 0.05, ^**^*P* < 0.01, ^***^*P* < 0.001..(GD=gestation day, DPP=day post-partum, VAD=vitamin A deficiency, VAS=Vitamin A sufficient, VA-vitamin A)

**Table S1.** Antibodies used in flow cytometry for NK cell, DC and T cell staining

|  | **Marker** | **Fluorochrome^*^** | **Antibody/Vendor/Cat. #** | **Isotype** | **Note** |
| --- | --- | --- | --- | --- | --- |
| NK cell markers | CD16 | FITC | Mouse Anti-porcine/Biorad/MCA1971F | IgG1 |  |
|  | SWC3aB | No | Mouse anti-porcine/S. Biotech | - |  |
|  | Streptavidin | APC | BD pharmigen/554067 | Streptavidin APC | Secondary antibody for SWC3a Biotin |
| DC subset markers | SWC3a (porcine) | PE | Mouse Anti-porcine Monocyte/Granulocyte-PE/Southern Biotech/ 4525-09 | IgG1 | Human analog CD172 |
|  | CD4 (porcine) | SPRD | Mouse Anti-porcine CD4a/Southern Biotech/4515-13 | IgG2b |  |
|  | CD11R1 (porcine) | No | Mouse Anti-pig CD11R1/Serotec/MCA1220 | IgG1 | Human analog CD11b |
|  | CD103 (human) | FITC | Mouse Anti-Pig CD103 FITC Conjugated, clone B-Ly7/eBiosciences/11-1038-73 | IgG1 | Cross reacts with porcine CD103 |
|  | Class II | FITC | Mouse anti-pig/Biorad/MCA2314F | IgG1 |  |
| T cell markers | CD3e | No | Mouse anti pig S. Biotech/4510-01 | - |  |
|  | CD4-Biotin | No | Mouse anti pig S. Biotech/ 4515-08 | - |  |
|  | CD25 | No | Mouse anti pig/Biorad/MCA1736GA | - |  |
|  | CD8 | FITC | S. Biotech/4520-02 | IgG2a |  |
|  | IFN-γ | PE | BD/559812 | IgG1 |  |
|  | Streptavidin-PE Cy7 | PE-Cy7 | BD/551419 | Streptavidin-PE Cy7 | Secondary Ab for CD4-Biotin/CD8 Biotin |
|  | IgG1 | APC | Rat anti Mouse IgG1-APC BD/550874 | Rat anti Mouse IgG1 | Secondary Ab for CD3e |
|  | CD4 | PE | BD/559586 | IgG2b |  |
|  | CD8 Biotin | No | Mouse anti pig/ S. Biotech/4520-08 | - |  |
|  | FOXP3 | FITC | Mouse anti-porcine Invitrogen/11-5773-82 | IgG1 |  |

**Table S2.** Primers used in qRT-PCR for gene expression analysis in ileum and mesenteric lymph nodes total RNA. GAPDH was used as the house keeping gene.

| **Gene** | **Sequence** | **Annealing Temperature** | **Product Size (bp)** |
| --- | --- | --- | --- |
| pIgR | Sense- AGCCAACCTCACCAACTTCC | 62°C | 140 |
|  | Antisense- CTGCTAATGCCCAGACCAC |  |  |
| RPB4 | Sense- GCAAGATGGAATGGGTTTG | 60°C | 82 |
|  | Antisense-GTTCTCTTTGACTCGGAAGCTG |  |  |
| RARα | Sense- AGCTGGGCAAATACACTACGAA | 55°C | 166 |
|  | Antisense- GGCAGCYGCTTGGCRAACTC |  |  |
| MAdCAM-1 | Sense- AGCCTGGGCTCCGTAAAGTC | 60°C | 155 |
|  | Antisense- TGGTCAGGGAAGGCGAACAC |  |  |
| VCAM-1 | Sense- CCCAAGGACCCAGTTATATC | 52°C | 104 |
|  | Antisense- GAAATCTGTGGAGCTGGTAG |  |  |
| GAPDH | Sense- CTTCACGACCATGGAGAAGG | 63°C | 170 |
|  | Antisense-CCAAGCAGTTGGTGGTACAG |  |  |
|  |  |  |  |
|  |  |  |  |
